# Investigation of Antioxidant/Oxidant Status and Antimicrobial Activities of *Lentinus tigrinus*

**DOI:** 10.1155/2018/1718025

**Published:** 2018-11-01

**Authors:** Mustafa Sevindik

**Affiliations:** Department of Biology, Faculty of Science, Akdeniz University, Antalya, Turkey

## Abstract

In the present study, antioxidant and antimicrobial potential of the *Lentinus tigrinus* (Bull.) Fr. mushroom was determined. Total antioxidant status (TAS), total oxidant status (TOS), and oxidative stress index (OSI) of the mushroom were measured with Rel Assay kits. Antimicrobial activities were tested on 9 standard bacterial and fungal strains (*Staphylococcus aureus*, *Staphylococcus aureus* MRSA, *Enterococcus faecalis*, *Escherichia coli*, *Pseudomonas aeruginosa*, *Acinetobacter baumannii*, *Candida albicans*, *Candida krusei*, and *Candida glabrata*) with a modified agar dilution method. It was determined that the TAS value of *L. tigrinus* was 1.748 ± 0.071, TOS value was 19.294 ± 0.237, and OSI was 1.106 ± 0.031. It was also found that mushroom extracts generally exhibited higher activity on *Candida albicans*, *C. krusei*, and *C. glabrata*. In conclusion, it was suggested that *L. tigrinus* can be used as a natural source due to its antioxidant and antimicrobial activities.

## 1. Introduction

Since historical times, several mushroom species have been consumed as nutrients and medicine of natural origin by humans [1]. Mushrooms can be considered as functional nutrients due to their health benefits and nutritional properties. In recent years, functional nutrients were again the center of focus for the consumers whose interest in human health, nutrition, and prevention from diseases has increased [2, 3]. Previous studies on mushrooms reported that mushrooms possessed several medical properties such as antioxidant, antimicrobial, DNA-protective, analgesic, anti-inflammatory, cytotoxic, antiviral, anticancer, antiparasitic, immunomodulation effects, and hepatoprotective activity [4–18]. The identification of medical potential of the mushrooms is significant for identification of new natural resources for fighting the diseases.

*Lentinus tigrinus* is a wood-rotting basidiomycete with leathery flesh, strong aroma, and taste that makes it applicable in gourmet preparations [19]. This basidiomycetous mushroom is often seen growing on fallen logs in the forest from May to September [20]. Previous studies reported that this mushroom contains high amounts of carbohydrates, proteins, fibers, and minerals [19].

The present study aimed at determining the total antioxidant status, total oxidant status, and oxidative stress index of *L. tigrinus* (Bull.) Fr. mushroom and the antimicrobial activities of the ethanol (EtOH), methanol (MeOH), and dichloromethane (DCM) extracts of the mushroom. This study will evaluate the availability of *L. tigrinus* mushroom for pharmacological designs.

## 2. Materials and Methods

### 2.1. Laboratory Studies

*Lentinus tigrinus* (Bull.) Fr. study samples were collected in Gaziantep province, Turkey. Morphological (shape, color, and size) and ecological characteristics of the samples were recorded in the field conditions. The microscopic characteristics of the specimens transported to the laboratory under appropriate conditions were determined by light microscopy using a 3% KOH solution (Leica DM750). The specimen was identified morphologically using the references of Käärik [21], Knudsen [22], Bresadola [23], Dähncke [24], Roux [25], and Boccardo et al. [26]. After the collected mushroom samples were identified, they were dried at 40°C in an incubator. Then, they were pulverized in a mechanical grinder. Then, pulverized 30 g mushroom samples were placed in cartridges, and the extracts were obtained with ethanol (EtOH) (Merck), methanol (MeOH) (Merck), and dichloromethane (DCM) (Merck) in a soxhlet extractor (Gerhardt EV) at 50°C for approximately 6 hours. The extracts were then concentrated under pressure at 40°C in a rotary evaporator (Heidolph Laborota 4000 Rotary Evaporator) to conduct the tests at +4°C [1].

### 2.2. Determination of Total Antioxidant Status (TAS), Total Oxidant Status (TOS), and Oxidative Stress Index (OSI)

The mushroom total antioxidant status (TAS), total oxidant status (TOS) levels, and oxidative stress index (OSI) were determined with Rel assay brand commercial kits (Rel Assay Kit Diagnostics, Turkey). Trolox was used as the calibrator in the TAS tests and hydrogen peroxide in the TOS tests [27, 28]. To determine the OSI, the mmol unit of TAS and the *μ*mol unit of the TOS were cross-converted and the index value was expressed as percentage [28]. The TAS and TOS tests were conducted on 5 mushroom samples in 5 replicates.

### 2.3. Antimicrobial Activity Tests

Antimicrobial activity tests were conducted with the agar dilution method recommended by the Clinical and Laboratory Standards Institute (CLSI) and the European Committee on Antimicrobial Susceptibility Testing (EUCAST) on mushroom EtOH, MeOH, and DCM extracts. Minimal inhibitor concentration (MIC) for each extract was determined against standard bacterial and fungal strains. *Staphylococcus aureus* ATCC 29213, *Staphylococcus aureus* MRSA ATCC 43300, and *Enterococcus faecalis* ATCC 29212 were used as Gram-positive bacteria. *Escherichia coli* ATCC 25922, *Pseudomonas aeruginosa* ATCC 27853, and *Acinetobacter baumannii* ATCC 19606 were used as Gram-negative bacteria. *Candida albicans* ATCC 10231, *Candida krusei* ATCC 34135 ATCC 13803, and *Candida glabrata* ATCC 90030 were used as fungi. Bacterial strains were precultured in Muller-Hinton Broth medium, and fungal strains were precultured in the RPMI 1640 broth medium. To obtain a standard inoculum, the turbidity of the bacteria and fungi was designed based on the McFarland 0.5 scale. All extracts were tested at concentrations of 800-12.5 *μ*g/mL, and all dilutions were prepared with distilled water. Solvents used for the extraction were also tested for antimicrobial activity. Fluconazole and amphotericin B were used as reference drugs for the fungi and amikacin, and ampicillin and ciprofloxacin were used as reference drugs for the bacteria. The minimal dilution that inhibited the growth of bacteria and fungi was identified as the minimum inhibitory concentration (MIC) [29–34].

## 3. Results and Discussion

### 3.1. Total Antioxidant Status (TAS), Total Oxidant Status (TOS), and Oxidative Stress Index (OSI)

The mushroom TAS, TOS, and OSI values were determined with the Rel Assay kits. The findings demonstrated that the *L. tigrinus* TAS value was 1.748 ± 0.071, TOS value was 19.294 ± 0.237, and OSI was 1.106 ± 0.031. Mushrooms have the potential to contain several antioxidant enzymes and reduced coenzymes and reduced molecules such as phenolic compounds that include electron sources with the antioxidant effect. The analysis and evaluation of TAS as a marker of the system that reflects the whole of the enzymatic and nonenzymatic molecules that the fungi potentially produce and maintain are significant in identification and determination of new natural antioxidant sources. There are no previous studies that aimed at determining TAS, TOS, and OSI of *L. tigrinus*. In previous studies conducted with mushrooms on oxidative stress, it was determined that TAS values of *Omphalotus olearius* and *Paxillus involutus* were 2.827 and 1.230, TOS values were 14.210 and 7.533, and OSI values were 0.503 and 0.613, respectively [35, 36]. It was also reported that the TAS values of *Helvella leucomelaena* and *Sarcosphaera coronaria* were 2.367 and 1.066, TOS values were 55.346 and 41.662, and OSI values were 2.338 and 3.909, respectively [37]. In other studies, it was determined that the TAS value of *Pleurotus eryngii* was 1.93, and the TAS value of *Auricularia polytricha* was 0.93 [38, 39]. In the present study, it was observed that the TAS value of *L. tigrinus* used in this study was higher when compared to *P. involutus*, *S. coronaria*, and *A. polytricha* mushroom and lower when compared to *O. olearius*, *H. leucomelaena*, and *P. eryngii*. It was also observed that *L. tigrinus* TOS and OSI values were higher when compared to *P. involutus* and *O. olearius* and lower when compared to *H. leucomelaena* and *S. coronaria*. It was considered that the difference among the mushrooms was due to the variation in their capacity for reactive oxygen species production as a result of the environmental factors in fungal habitats. Thus, it is suggested that *L. tigrinus* had antioxidant potential; however, due to its high oxidant compound production capacity, the samples collected in Gaziantep province should not be consumed in excess. Furthermore, it was determined that samples collected in regions with adequate mushroom oxidative stress levels can be consumed as a natural antioxidant source.

### 3.2. Antimicrobial Activity

It was reported that mushrooms produce a variety of biologically active compounds, often associated with the cellular wall, and it was determined that several such compounds have biological activities. Indigenous communities considered mushrooms as potential sources of antibacterial drugs, and antibiotic research were initially started and succeeded with mushrooms [4, 40]. Thus, identification of fungal antimicrobial activities is very important for identification of the new antibacterial and antifungal agents. In the present study, EtOH, MeOH, and DCM extracts of *L. tigrinus* were evaluated against *S. aureus*, *S. aureus* MRSA, *E. faecalis*, *E. coli*, *P. aeruginosa*, *A. baumannii*, *C. albicans*, *C. krusei*, and *C. glabrata*. *L. tigrinus* extracts were compared with ampicillin, amikacin, ciprofloxacin, fluconazole, and amphotericin B which were used to treat general bacterial and fungal infections. In particular, *L. tigrinus* extracts showed antibacterial activity to different widths depending on the type of the infectious agent. The findings are presented in Table 1.

Antimicrobial activity test findings demonstrated that EtOH extracts generally exhibited higher levels of activity on test microorganisms. Table 1 shows that the mushroom extracts were not effective on *A. baumannii*. Furthermore, mushroom EtOH extract exhibited activity against *E. coli* and *P. aeruginosa*, while MeOH and DCM extracts did not exhibit any activity in tested concentrations. It was found that the mushroom extracts were generally more active on fungal strains. Previous studies that were conducted to determine the antimicrobial activities of *L. tigrinus* reported that the acetonitrile extract was active against *E. coli* and *S. aureus* [20]. In a separate study, it was determined that water and *n*-hexane extracts of *L. tigrinus* were active against *E. coli*, *Bacillus subtilis*, *B. licheniformis*, *S. aureus*, and *Agrobacterium tumefaciens* in various concentrations [41]. Furthermore, it was determined that mushroom extracts were active on *S. aureus*, *S. aureus MRSA*, *E. faecalis*, *C. albicans*, *C. glabrata*, and *C. krusei* in concentrations of 100–800 *μ*g/mL. In conclusion, it was determined that *L. tigrinus* can be consumed as a natural antimicrobial source against the microorganism that demonstrated the abovementioned activities. Mushrooms contain many compounds that show antimicrobial and antioxidant effects. In future, GC-MS studies can identify compounds in *L. tigrinus.* These compounds can be isolated and identified as compounds that cause antimicrobial and antioxidant effects. Crude extracts of *L. tigrinus* were used in our study. Antioxidant and antimicrobial potential of *L. tigrinus* was determined.

## 4. Conclusions

In the present study, total antioxidant status, total oxidant status, oxidative stress index, and antimicrobial potential of *L. tigrinus* were determined. It was determined that the mushroom possessed antioxidant potential as a result of the conducted analyses. However, it was recommended to limit the consumption of this mushroom due to high oxidant values. It was determined that *L. tigrinus* mushroom collected in regions with adequate oxidative stress levels may be consumed as a natural antioxidant source. Furthermore, the present study demonstrated that *L. tigrinus* may serve as a natural antimicrobial source against the microorganisms that exhibited activity in the tests conducted in the study.

## Figures and Tables

**Table 1:** Minimum inhibitory concentrations of different extracts of *L. tigrinus* and standard antibiotics against test microorganisms.

	*S. aureus*	*S. aureus* MRSA	*E. faecalis*	*E. coli*	*P. aeruginosa*	*A. baumannii*	*C. albicans*	*C. glabrata*	*C. krusei*
EtOH	200	200	200	800	800	None	400	100	200
MeOH	200	400	200	None	None	None	800	200	200
DCM	800	800	800	None	None	None	800	400	400
Ampicillin	1.56	3.12	1.56	3.12	3.12	—	—	—	—
Amikacin	—	—	—	1.56	3.12	3.12	—	—	—
Ciprofloxacin	1.56	3.12	1.56	1.56	3.12	3.12	—	—	—
Fluconazole	—	—	—	—	—	—	3.12	3.12	—
Amphotericin B	—	—	—	—	—	—	3.12	3.12	3.12

The MIC values are presented in units of *µ*g/mL.

## Data Availability

No data were used to support this study.
